# The Relationship Between the Clavicular Tilt Angle and Sagittal Spinal Alignment Associated With Hyperkyphosis Posture

**DOI:** 10.7759/cureus.82044

**Published:** 2025-04-10

**Authors:** Wataru Nukii, Takahiro Fukazawa, Yukihiro Higo, Rio Yamanaka, Ami Uchino, Naoto Kato, Natsume Tsubaki, Kazuyoshi Nakanishi, Hirokatsu Sawada, Masachika Niimi

**Affiliations:** 1 Rehabilitation Medicine, Nihon University Graduate School of Medicine, Tokyo, JPN; 2 Rehabilitation Medicine, Nihon University School of Medicine, Tokyo, JPN; 3 Rehabilitation, Nihon University Itabashi Hospital, Tokyo, JPN; 4 Orthopaedic Surgery, Nihon University School of Medicine, Tokyo, JPN

**Keywords:** clavicle, kyphosis, lumbar lordosis angle, sagittal vertical axis, thoracic kyphosis

## Abstract

Introduction

Hyperkyphosis is a deformity of the spine’s forward curvature in the sagittal alignment, and its indices include the sagittal vertical axis (SVA), thoracic kyphosis (TK), and lumbar lordosis angle (LLA). These indices are difficult to evaluate in patients who cannot maintain a standing position. Therefore, we sought to determine whether the mean clavicular tilt angle, which can be evaluated regardless of the patient's posture, can be used as an indicator of hyperkyphosis. We hypothesized that the mean clavicular tilt angle would correlate with established sagittal spinal alignment parameters and could serve as a reliable surrogate indicator of hyperkyphosis in patients unable to stand. Hyperkyphosis can be evaluated using the indices that require standing or sitting. However, no studies have attempted to evaluate hyperkyphosis regardless of the patient's posture.

Methods

This retrospective study analyzed 106 patients (aged ≥65 years) who underwent surgery for lumbar spinal canal stenosis at our hospital between March 2023 and July 2024. All patients had both a plane radiography of the frontal plane including the clavicle and a plane radiography of the entire spine in the sagittal plane from the cervical spine to the pelvis taken before surgery. The mean clavicular tilt angle was measured using two methods: the conventional method and a newly devised method. The new method modifies the angle reference point to enhance applicability in cases with extreme postural deformities. The correlation between the clavicular tilt angle and SVA, TK, and LLA was examined for each method.

Results

A positive correlation (r = 0.343, p < 0.05) was found between the mean clavicular tilt angle and TK using the conventional method. A positive correlation (r = 0.562, p < 0.05) was found between the mean clavicular tilt angle and SVA using the new method, and a negative correlation (r = -0.437, p < 0.05) was found between the mean clavicular tilt angle and LLA using the new method, indicating moderate to strong correlations with spinal alignment parameters.

Conclusions

This study suggests that the mean clavicular tilt angle may be a useful indicator of hyperkyphosis. In particular, the new measurement method may serve as a practical tool for evaluating hyperkyphosis in frail or immobile populations. However, being a single-center retrospective study, these findings require validation through larger prospective studies.

## Introduction

Hyperkyphosis is a postural change that is often seen in the elderly and presents with changes in the alignment of the thoracolumbar spine and a forward-bent trunk posture [1,2]. The prevalence of hyperkyphosis increases with age, especially after the age of 40, and is 20 to 40% in adults over the age of 60 [3]. In addition to the general decline in muscle strength and exercise tolerance associated with aging, hyperkyphosis is also considered to affect physical functions such as a decline in walking and balance abilities due to reduced spinal mobility and trunk function [4,5]. Especially for the elderly with hyperkyphosis, the risk of falling is higher due to poor upright posture and decreased balance ability [2]. In addition, there is a tendency for people affected by hyperkyphosis to be less active, and it has been reported that their satisfaction with life and quality of life (QOL) decline due to the limited leisure activities [1,6]. Furthermore, hyperkyphosis can cause restrictive ventilatory impairment due to spinal deformity, and there are reports that this can lead to decreases in respiratory muscle strength, vital capacity, and lung and thorax compliance, which can affect survival time [7,8]. Advanced hyperkyphosis posture is thought to increase oxygen consumption during walking and cause deformation and reduced mobility of the rib cage, which limits lung capacity. Among patients with hyperkyphosis, the elderly and women in particular tend to have lower respiratory function, and the mortality rate from lung diseases such as pneumonia is reported to be higher [9-11].

Hyperkyphosis is a deformity of the spine’s forward curvature in the sagittal spinal alignment, and there are several indices known to be related to the degree of this curvature deformity. One of them is the sagittal vertical axis (SVA), which indicates the degree of forward displacement of the spinal center of gravity in a standing position. The SVA indicates the degree of forward displacement of the center of gravity by measuring the distance from the plumb line dropped from the center of the seventh cervical vertebral body to the posterosuperior corner of the sacrum vertebral body based on a standing spine radiography of the sagittal plane. SVA is also known to increase significantly in patients with sarcopenia [12]. In addition, there are other indices such as thoracic kyphosis (TK) and lumbar lordosis angle (LLA). TK is an index that expresses hyperkyphosis of the thoracic vertebrae [13]. LLA is an index of lumbar lordosis, and when lumbar lordosis disappears, hyperkyphosis progresses. LLA is correlated with postural instability and the tendency to fall [14].

Evaluating hyperkyphosis is generally difficult in patients who cannot maintain a standing position, but if the evaluation is of the alignment that can be measured on the body surface or chest, measurement is possible regardless of whether the patient can keep a standing position. The clavicular tilt angle in particular is easy to measure using plain chest radiography. High correlations between the clavicular tilt angle measured using a goniometer from the body surface and those measured using chest radiography, as well as the feasibility and effectiveness of the goniometer method have been reported [15,16]. The clavicular tilt angle has already been shown to be useful in predicting shoulder balance after surgery in patients with scoliosis by measuring the difference between the angles on the left and right sides [17,18]. As mentioned above, because hyperkyphosis has a negative impact on health, it is important to identify the condition from the early stages of rehabilitation intervention. If it is possible to predict the state of posture and physical function from the clavicular tilt angle, which can be evaluated based on plain chest radiography, it may be possible to use this to help determine the rehabilitation treatment and treatment goals in frail or immobile patients.

Patients with lumbar spinal stenosis often adopt a forward-leaning posture to alleviate neural compression [19]. Therefore, this study focused on lumbar spinal canal stenosis, which is likely to have a high prevalence of hyperkyphosis. 

The current study aimed to clarify the relationship between clavicular tilt angle and sagittal spinal alignment associated with hyperkyphosis posture.

## Materials and methods

Ethics statement

This study was conducted in compliance with the principles of the Declaration of Helsinki and approved by the Nihon University Itabashi Hospital Certified Clinical Research Review Board (RK-250204-1). Informed consent was obtained through an opt-out process, and the need for written informed consent was waived owing to the retrospective nature of this study.

Study design

**Figure 1:**
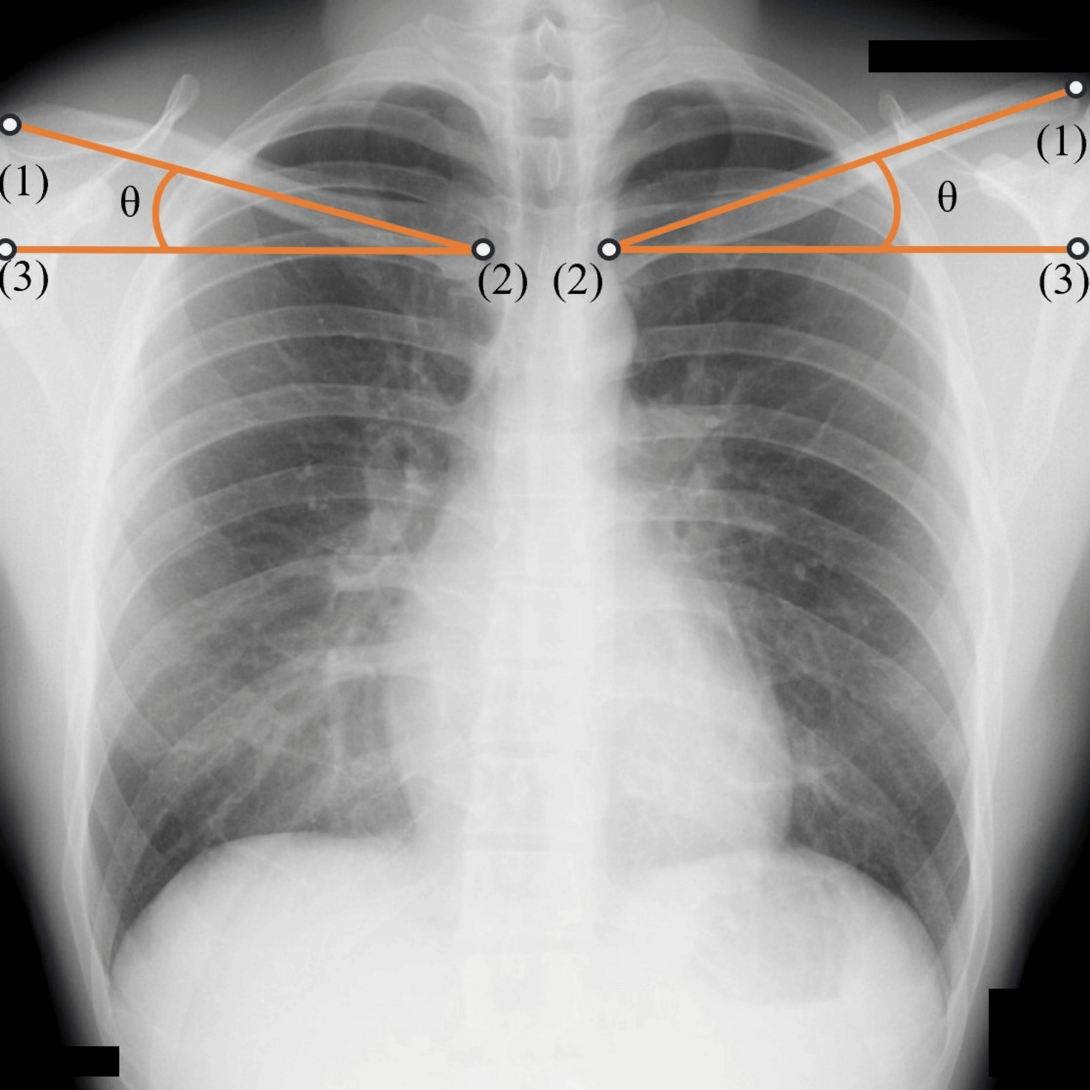
The existing method for measuring the clavicular tilt angle. (1): The midpoint of the acromial facet of the clavicle. (2): The midpoint of the sternal facet of the clavicle. (3): A point on the horizontal line drawn from the midpoint of the sternal facet of the clavicle. θ: Clavicular tilt angle

**Figure 2:**
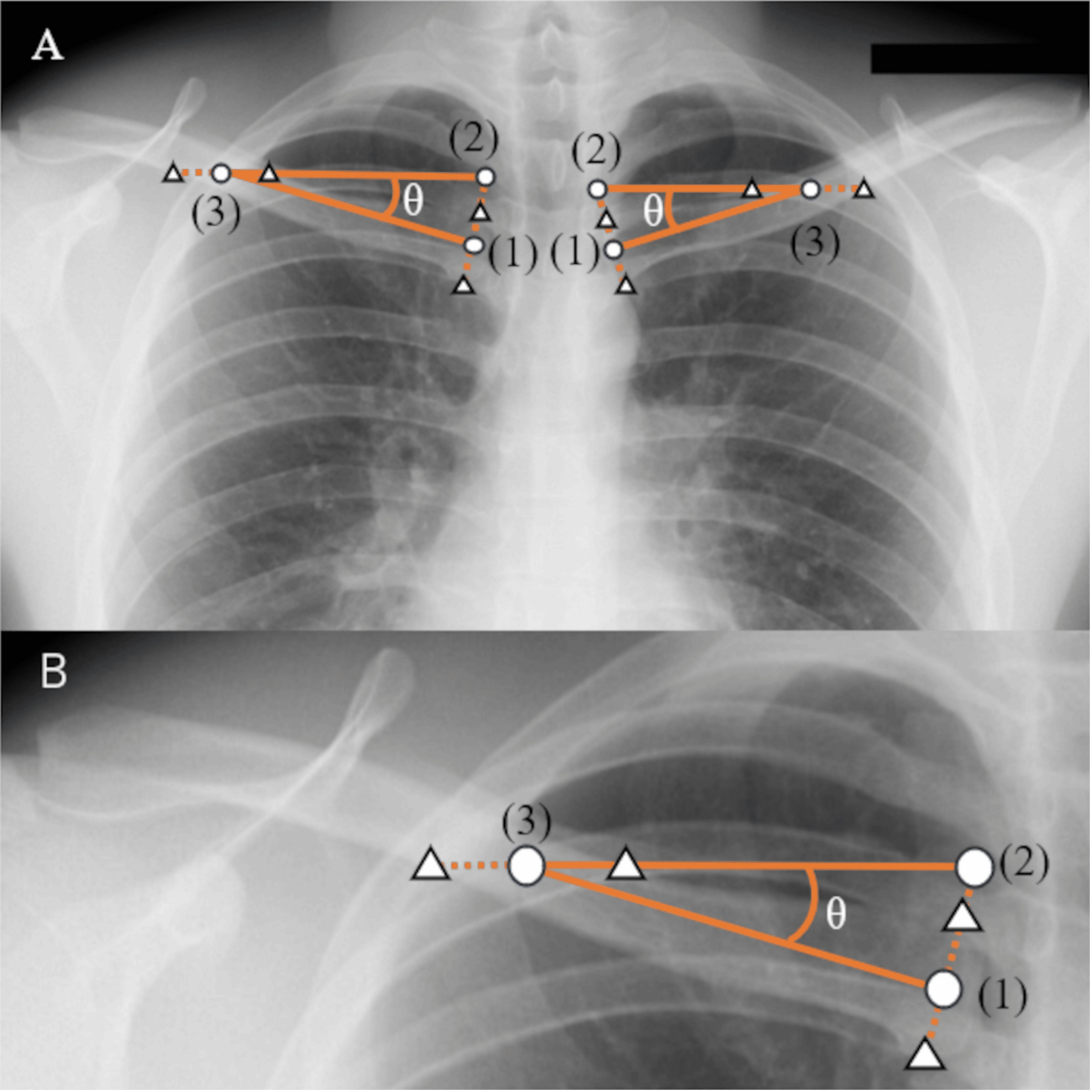
The new method for measuring the clavicular tilt angle. (A): The new method for measuring the clavicular tilt angle (B): Enlarged figure of (A) (1): The midpoint of the sternal facet of the clavicle. (2): The point where the line segment connecting the upper and lower edges of the sternal facet is extended 1.5 times upwards. (3): The midpoint of the line segment where the horizontal line drawn from (2) and the clavicle intersect. Δ: Points on the edge of the clavicle θ: Clavicular tilt angle

This retrospective study enrolled 106 patients (57 men and 49 women) who were 65 years or older and underwent surgery for lumbar spinal canal stenosis at our hospital between March 2023 and July 2024. The mean age was 76±6.22 years (range: 66-90 years). They had to have both a plain radiography of the frontal plane including the clavicle and a plain radiography of the entire spine in the sagittal plane from the cervical spine to the pelvis taken before the surgery. The chest X-ray was taken in the postero-anterior view in the standing position, at the same time as a plain radiography of the entire spine. Patients with a history of spinal fracture or surgery, patients for whom it was difficult to identify the reference vertebra, and patients for whom the clavicular tilt angle could not be measured were excluded. The clavicular tilt angle was measured using two methods: the existing method based on the methods described below by Noguchi et al., and a novel method devised for the current study. Patients for whom the clavicular tilt angle could not be measured using either method were excluded from the analysis pertaining to that measuring method. There were 46 and 50 patients in the existing method group and the novel method group, respectively. The clavicular tilt angle was measured using frontal plain chest radiography, and the SVA, TK, and LLA were measured using sagittal plain radiography of the entire spine. The clavicular tilt angle was first measured by referring to the methods of Noguchi et al. and calculating the angle formed by the line connecting the midpoints of the sternal and the acromial facets of the clavicle and the horizontal line (Figure 1) [20] (hereafter referred to as the “existing method”). Then, the original method for measuring the clavicular tilt angle was devised and measurements were taken. The angle between a horizontal line and a line connecting the midpoint of the sternal facet and the midpoint of a line drawn horizontally on plain radiography from the point where the line segment connecting the upper and lower edges of the sternal facet was extended upward 1.5 times to its intersection with the clavicle was measured (Figure 2) (hereinafter referred to as the “new method”). SVA was measured as the distance between the vertical line from the center of the seventh cervical vertebral body and the posterosuperior corner of the sacrum. TK was measured as the angle between the lower margin of the fourth thoracic vertebral body and the lower margin of the twelfth thoracic vertebral body, and LLA was measured as the angle between the upper margin of the first thoracic vertebral body and the upper margin of the sacrum.

All measurements were performed by the same examiner. For the clavicular tilt angle by the new method, a blind measurement by another examiner was performed in addition to the first measurement in order to evaluate the inter-rater reliability.

The normality of each index was confirmed using the Shapiro-Wilk test. Then, the correlation between the SVA, TK, and LLA was examined for the average clavicular angles measured by the existing method and the new method. The correlation coefficients between indices that showed normality were calculated using Pearson's correlation, and the correlation coefficients between indices that did not show normality were calculated using Spearman's rank correlation. The intraclass correlation coefficient (ICC) (2,1) was used to evaluate the inter-rater reliability of the clavicular tilt angle measured using the new method to confirm the reproducibility of the measurement. For the 35 cases where the average clavicular tilt angle was obtained by both the existing and the new methods, a paired t-test was performed and correlation coefficient were calculated for the two methods. EZR Version 1.68 was used for statistical analyses, and the significance level was set at 5%.

## Results

The normality of each index was confirmed with the Shapiro-Wilk test. Normality was observed in the mean clavicular tilt angle by the existing method and TK and LLA. Normality was not observed for the mean clavicular tilt angle by the new method and SVA.

**Figure 3:**
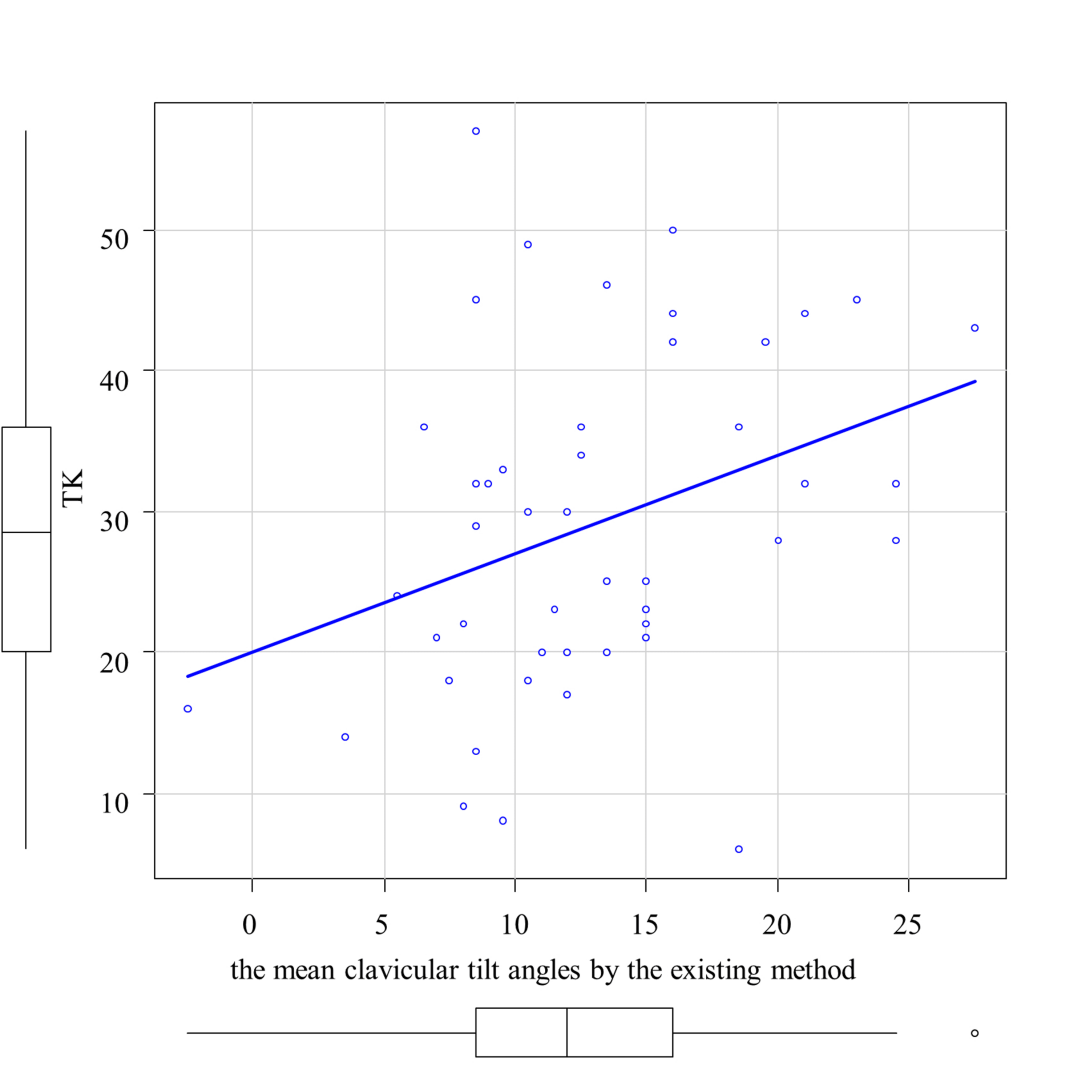
Correlation between the mean clavicular tilt angles by the existing method and thoracic kyphosis (TK)

**Figure 4:**
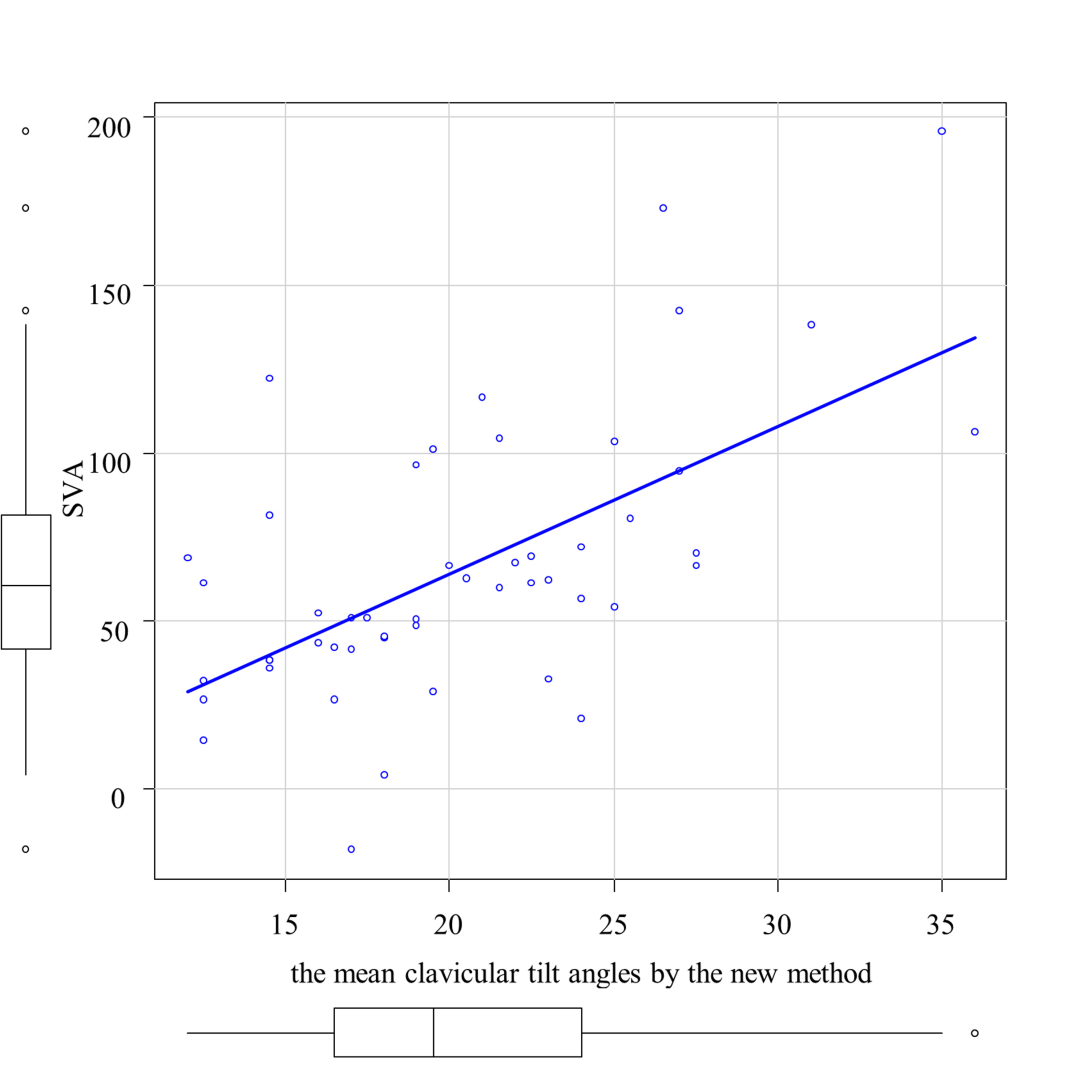
Correlation between the mean clavicular tilt angles by the new method and sagittal vertical axis (SVA)

**Figure 5:**
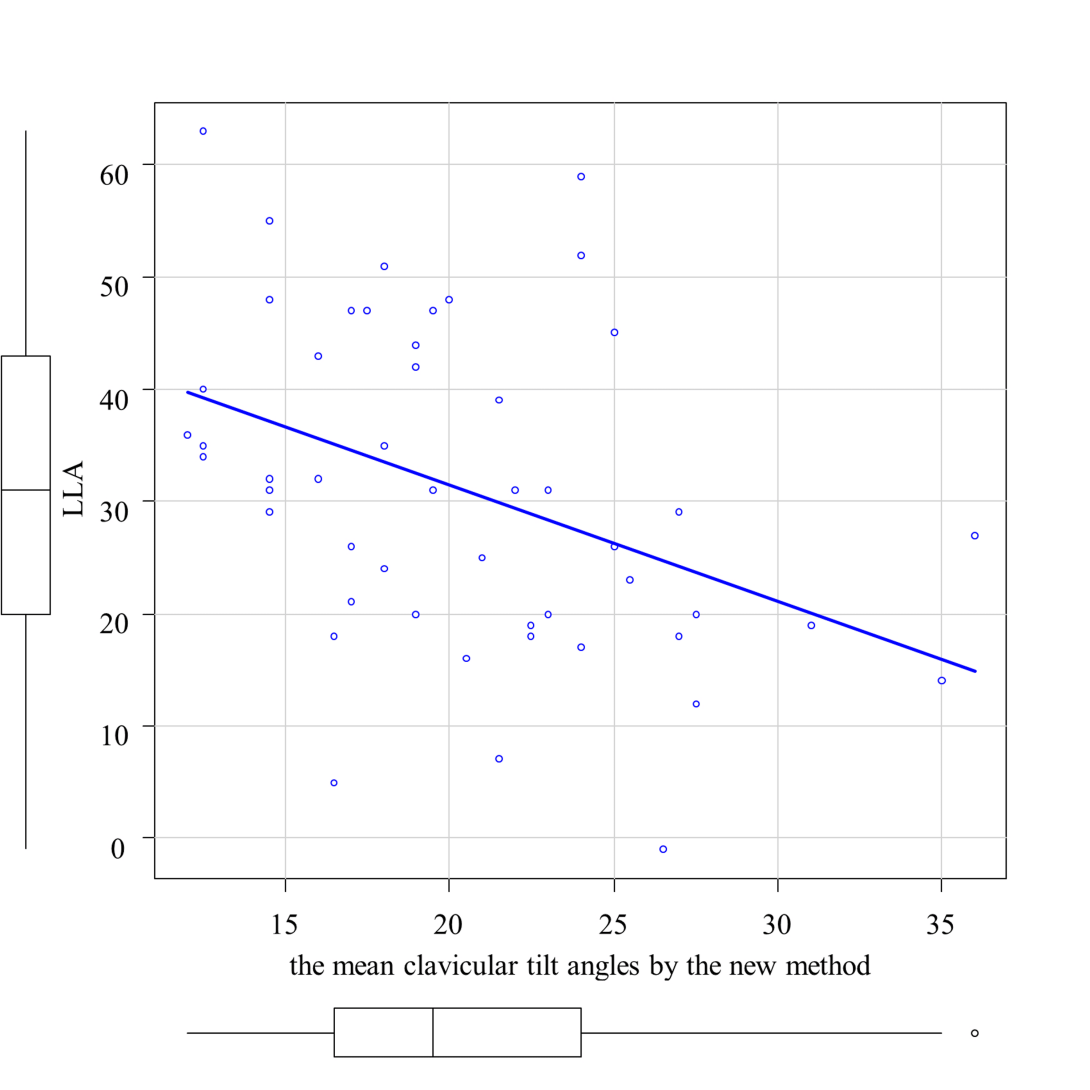
Correlation between the mean clavicular tilt angles by the new method and lumbar lordosis angle (LLA)

The correlation analysis between the average clavicular tilt angle by the existing method and TK is shown in Figure 3. A significant correlation was observed between the mean clavicular tilt angle by the existing method and TK, with a correlation coefficient of 0.343 (p < 0.05) (Table 1). Then, the correlation analyses between the average clavicular tilt angle by the new method and SVA and LLA, respectively, are shown in Figure 4 and Figure 5. A significant correlation was observed between the mean clavicular tilt angle by the new method and SVA, and between the mean clavicular tilt angle by the new method and LLA, with correlation coefficients of 0.562 (p < 0.05) and -0.467 (p < 0.05), respectively (Table 2). Of the 19 cases for which the mean tilt angle could not be calculated by the existing method because the acromial facet could not be identified, the angle was successfully calculated for 14 cases by the new method.

**Table 1:** Correlation coefficients of the mean clavicular tilt angles by the existing method A p-value of <0.05 was considered statistically significant, whereas a p-value >0.05 was considered statistically insignificant. SVA: sagittal vertical axis, TK: thoracic kyphosis, LLA: lumbar lordosis angle

	Correlation coefficients	p-value	*:p<0.05
SVA	0.219	0.14	
TK	0.343*	0.02	
LLA	0.0489	0.75	

**Table 2:** Correlation coefficients of the mean clavicular tilt angles by the new method A p-value of <0.05 was considered statistically significant, whereas a p-value >0.05 was considered statistically insignificant. SVA: sagittal vertical axis, TK: thoracic kyphosis, LLA: lumbar lordosis angle

	Correlation coefficients	p-value	*:p<0.05
SVA	0.562*	<0.001	
TK	-0.0479	0.74	
LLA	-0.437*	0.002	

**Figure 6:**
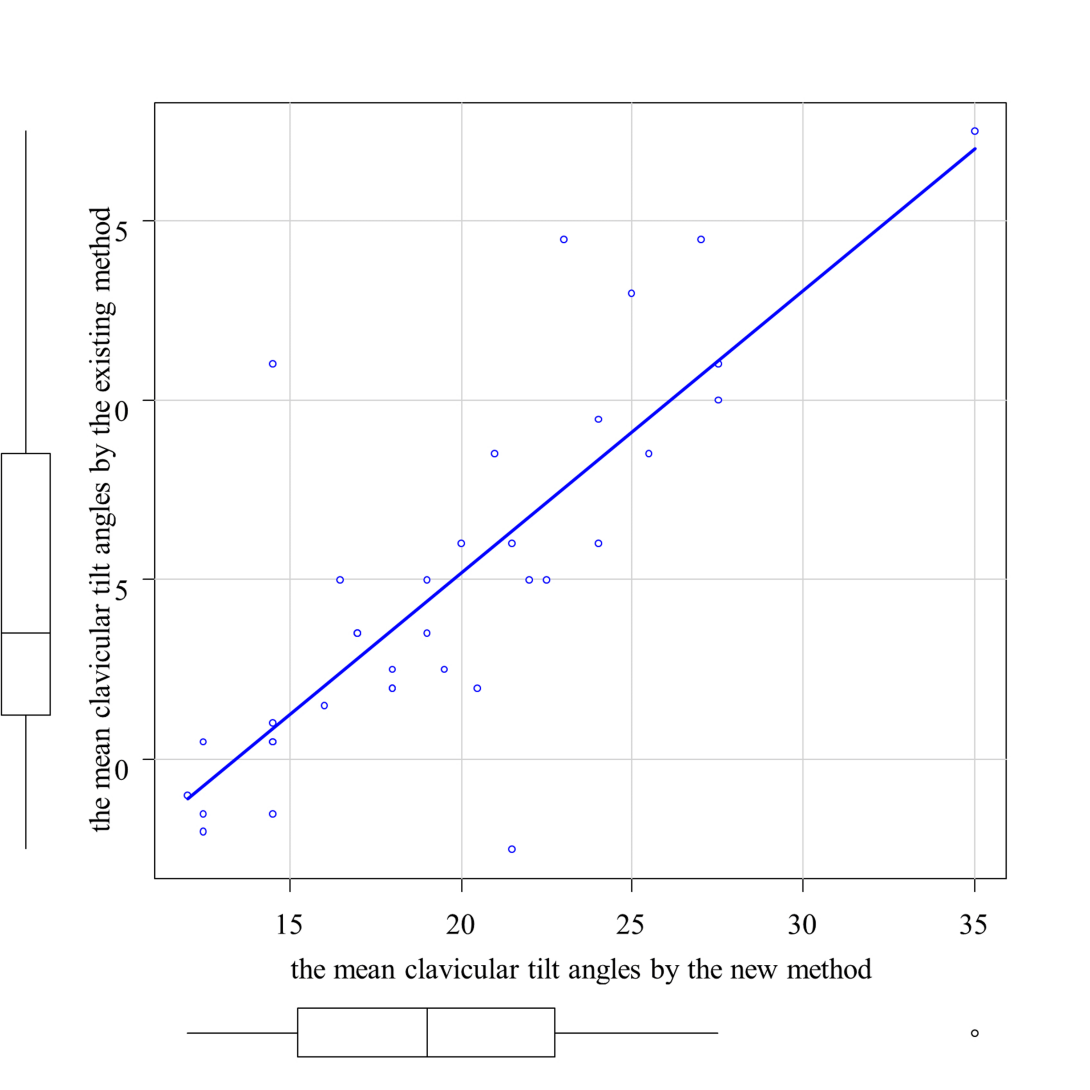
Correlation between the mean clavicular tilt angles by the new method and the existing method

The ICC (2,1) for the mean clavicular tilt angle by the new method was 0.869 (p < 0.05), indicating good inter-rater reliability. For the 35 cases for which both the existing and new methods were used, the mean clavicular angle by the existing method was 14.9 ± 5.2 (mean ± standard deviation), and the mean clavicular angle by the new method was 19.6 ± 5.3. The paired t-test showed a significant difference (p < 0.05). Correlation analysis between the existing and new methods is shown in Figure 6. The correlation coefficient was 0.800 (p < 0.05).

## Discussion

The current study showed that the average clavicular tilt angle measured by the new method positively correlated with SVA, while the average clavicular tilt angle measured by the new method negatively correlated with LLA. Moreover, the usefulness of the new method of measuring the mean clavicular tilt angle was demonstrated in addition to the existing method.

An increase in SVA above 40 mm has a negative impact on the patient's physical function and activities of daily living (ADLs). A target value of 40 mm or less is recommended for the SVA to maintain a good quality of life [21]. It has been reported that the group with SVA of 40 mm or more had weaker back extensor strength and a smaller skeletal muscle mass than the group with SVA of less than 40 mm [22]. It has also been reported that falls increased when SVA was 40 mm or greater [23]. In addition, there was a report that increased SVA correlated with worsening scores on the SF-12, an indicator of the ADLs [24].

Decreased LLA has a negative impact on the patient's physical function and ADLs. The concept of locomotive syndrome is defined as a condition in which walking ability and ADLs are limited due to dysfunction of one or more of locomotive organs including muscles, bones, joints, cartilage, and intervertebral discs [25], and it has been reported that as LLA decreased, the degree of locomotive syndrome increased as indicated by the three stages based on the progression [26]. Moreover, it has been reported that LLA is inversely correlated with performances on maximal walking speed, 10-meter walk test, Timed Up and Go Test, six-minute walk test, and one-leg standing time [27].

Patients with hyperkyphosis are likely to have a higher mean clavicular tilt angle. It has been reported that patients with hyperkyphosis have increased tension in the upper part of the trapezius muscle [28]. It has also been reported that excessive contraction of the upper part of the trapezius muscle increased the clavicular tilt angle [29]. Since patients with hyperkyphosis have higher TK and SVA and lower LLA [12-14], the results of the current study that showed correlation between the mean clavicular tilt angle with TK and SVA and inverse correlation with LLA are also in line with the literature. The difference between the existing method and the new method in the indices of spinal alignment that showed significant correlation may be due to the small number of cases and selection bias caused by differences in posture at the time of imaging. As mentioned above, hyperkyphosis causes adverse health effects in many ways, and the mean clavicular tilt angle could be an index for those effects.

The existing method could not be calculated in some cases where the mean clavicular tilt angle was high. Of the 65 patients who were included in the study, excluding those who met exclusion criteria other than the mean clavicular tilt angle itself, 19 patients had difficulty identifying the acromial facet of the clavicle. In cases with a high mean clavicular tilt angle, the clavicle acromial facet which serves as the angle reference, was located outside of the lung field. It was outside the field of view of the plain chest radiography. This is thought to be the cause of the difficulty in identifying the acromial facet.

The new method enabled the calculation of the angle even in cases with a high mean clavicular tilt angle. The angle reference was not the clavicle acromial facet. Instead, it was the midpoint of a horizontal line segment. This line was drawn from a point where the line segment connecting the upper and lower edges of the sternal facet was extended 1.5 times upward. The line extended to the intersection with the clavicle. As a result, the mean clavicular tilt angle could be calculated without using the clavicle acromial facet, and the mean tilt angle could also be calculated for cases with a higher mean clavicular tilt angle. As a result, the mean clavicular tilt angle could be calculated without using the clavicle acromial facet. The mean tilt angle could also be calculated for cases with a higher mean clavicular tilt angle.

The new method is expected to be useful as a measurement method. The results of ICC (2,1) indicate high inter-rater reliability. In addition, a higher correlation was observed in comparison with the existing method. On the other hand, in the 35 cases in which the mean clavicular tilt angle could be calculated by both the existing and new methods, the mean clavicular tilt angle by the new method was 4.7º greater than by the existing method. This is likely because the clavicle has an S-shaped curve structure and the curve is caudally convex on the sternal end [30], and the clavicular mean tilt angle for the new method is calculated to be higher. Plain chest radiography for the purpose of imaging the lung fields can be used to calculate the mean clavicular tilt angle in many cases, which may be useful as an indicator to predict skeletal muscle mass, fall risk, and ADLs as mentioned above.

This study was conducted as a single-center, retrospective study. In measuring the mean clavicular tilt angle using the existing method, there were many patients with a large clavicular tilt angle whose acromial facet of the clavicle could not be identified by plain chest radiography as it fell outside of the lung field. This was often the case in patients with a large clavicular tilt angle. It is also possible that the small number of cases (46 and 50, respectively for the existing and the new methods) did not allow for a significant correlation which was originally expected. In addition, the posture for the plain chest radiography was not standardized and may have contained selection bias. Furthermore, except for the additional measurement of the mean clavicular tilt angle by the new method for the ICC calculation, the same person performed the measurement for all the indices and statistical processing of each value, which may have contributed to information bias. Additionally, the chest X-ray was taken in the postero-anterior view in the standing position. However, since this was a retrospective study, it was not possible to standardize the detailed posture during imaging. Variations in posture among patients during imaging may have introduced information bias. Therefore, to eliminate this potential bias in future studies, prospective research is needed.

## Conclusions

The significant correlation between the mean clavicular tilt angle and hyperkyphosis indices such as TK, LLA, and SVA was demonstrated. It was suggested that the mean clavicular tilt angle may be useful as an indicator of hyperkyphosis. It is necessary to clarify the relationship between the mean clavicular tilt angle and ADLs in the future. Since this study was retrospective, prospective research is needed to eliminate selection and information biases and to further validate the findings.
